# Nutritional status of multiple sclerosis (MS) patients attending Kasr Alainy MS unit: an exploratory cross-sectional study

**DOI:** 10.1186/s42506-021-00080-3

**Published:** 2021-07-13

**Authors:** Zeinab E. Afifi, Rania I. Shehata, Asmaa F. El Sayed, El Sayed M. Hammad, Marwa R. Salem

**Affiliations:** 1 Public Health and Community Medicine Department, Faculty of Medicine, Cairo University, PO Box 109 El Malek El Saleh, Cairo, 11559 Egypt; 2 Department of Neurology, Faculty of Medicine, Cairo University, Cairo, Egypt; 3 Clinical Nutrition Department, National Nutrition Institute, Cairo, Egypt

**Keywords:** Nutritional assessment, Multiple sclerosis, Malnutrition, Fatigue, Exploratory study

## Abstract

**Background:**

Nutrition was claimed to be a factor in MS causation, course, complications, and management. Several studies were conducted to assess the nutritional status of MS patients; however, few studies were conducted to assess this problem in Egypt. Therefore, the purpose of the current study was to assess the nutritional status of a sample of MS patients.

**Methods:**

The researchers conducted an exploratory cross-sectional study among 76 relapsing-remitting MS (RRMS) patients attending Kasr Alainy Multiple Sclerosis Unit (KAMSU) from October 2018 to January 2019 to assess the nutritional status of a sample of MS patients. Data were collected using a structured interview questionnaire including an inquiry about the socioeconomic status, and nutritional status using anthropometric measurements, patient-generated subjective global assessment (PG-SGA), semi-quantitative food frequency questionnaires (SQFFQ), and hemoglobin level measurement. Assessment of fatigue was done using the Modified Fatigue Impact Scale 5-items version.

**Results:**

The mean age of the study participants was 30 ± 6 years. The disease duration ranged from 2 to 264 months. Malnutrition was prevalent among 67.1% (27.6 % overweight, 36.8% obese, and 2.6% underweight). Half of the investigated patients were anemic. According to the PG-SGA, more than half of the studied patients (53.9%) were classified as moderately or suspected malnourished. The unhealthy dietary habits such as taking only a few meals, junk food intake and skipping breakfast were observed in considerable proportions of the group. The SQFFQ revealed overconsumption of energy and fat, and less than acceptable consumption of dietary fibers by most of the studied patients.

**Conclusions:**

Overweight, obesity, anemia, and unhealthy dietary habits were prevalent among the RRMS patients attending the KAMSU. Nutrition care service is extremely needed for this group of patients.

## Background

Multiple sclerosis (MS) is a chronic inflammatory autoimmune disease of the central nervous system (CNS) [1]. The global median prevalence of MS was about 33/100,000 in 2013 [2]. The estimated prevalence of MS in Egypt was about 25/100.000 in different centers [3]. MS attacks women more than men; the prevalence ratio reaches 3.2:1, respectively [4]. MS is a leading cause of neurological disability in young adults. The social impact of the disability caused by MS is profound. It results in loss of employment and leads to dependency on care providers and social isolation [5].

The definite etiology of MS is unknown, but it is believed to be triggered by various environmental factors (unhealthy lifestyle with unhealthy dietary habits, vitamin D deficiency, childhood obesity, poisoning with heavy metals, cigarette smoking, and viral infections) in genetically susceptible individuals [6, 7].

MS patients have a double burden of malnutrition. Initially, overweight and obesity had a prevalence of more than 60% [8] due to immobility, steroid therapy, antidepressant drugs, and unhealthy dietary habits. When the disease advances, undernutrition, and cachexia occur [9]. Undernutrition exacerbates atrophy of limbs, so immobility develops. Respiratory muscle weakness occurs with an increased risk of infectio

ns, muscles of deglutition are affected, swallowing ability is impaired, and immunodeficiency occurs. Related infections are common causes of death [10, 11].

Hence, nutrition was claimed to be a factor in MS causation, course, complications, and management. Several studies were conducted to assess the nutritional status of MS patients. Apart from two studies conducted to address vitamin D deficiency and its effect on bone health and cognition in MS patients, few studies were conducted to assess this problem in Egypt [12, 13]. Therefore, the current study was conducted to assess the nutritional status of a sample of MS patients and to explore fatigue among this group of patients in Egypt.

## Methods

### Study setting and design

A cross-sectional study in which Remitting Relapsing Multiple sclerosis patients attending Kasr Alainy Multiple Sclerosis Research Unit (KAMSRU), in the Faculty of Medicine, Cairo University, were recruited over a period of 4 months from October 2018 to January 2019.

### Sample size and sampling technique

A representative sample size of 76 was calculated by Epi Info 7 Stat Calculator. Assumptions used are population size 96 (3 patients/day, 2 days/week for 16 weeks), prevalence of malnutrition among MS patients 60% [8], and confidence level of 95%. The inclusion criteria for enrollment were remitting relapsing MS patients (a study showed that relapsing-remitting MS (RRMS) subtype constitutes about 80 to 85% of all MS patients) [14], aged 20–40 years old (the onset of the disease is typically between ages 20 and 40 [14], and similar studies used the same age interval) [15]. Patients with metabolic diseases, mental, secondary progressive multiple sclerosis subtype, and those who refused to sign the informed consent were excluded.

### Data collection tools

The structured interview questionnaire comprised inquiry about each patient’s age, sex, socioeconomic status (El Gilany scale), nutritional status (the patient-generated subjective global assessment (PG-SGA), dietary habits questionnaire and, semi-quantitative food frequency questionnaire), and fatigue status (the Modified Fatigue Impact Scale 5-items version (MFIS-5)).

The socioeconomic status was assessed using a previously validated socioeconomic status scale proposed by El Gilany et al. [16]. This scale covers seven domains with a total score of 84 points, namely education, occupation, family (residence, and number), family possession, home sanitation including crowding index, economic, and healthcare domains (usual source of care). The socioeconomic status was classified according to the *quartiles* of the score into the following:

- Very low socioeconomic status, those with scores less than 21
- Low socioeconomic status, those with scores from 21 to less than 42
- Middle socioeconomic status, those with scores from 42 to less than 63
- High socioeconomic status, those with scores from 63 to 84 [16]

Assessment of nutritional status of the studied patients using the following:

1. *Anthropometric measurements*
  - Anthropometric measurements (namely; weight, height, and accordingly the body mass index (BMI)) were conducted for the enrolled patients. Participants were classified as normal weight if their BMI was between 18.5-24.9 kg/m2, overweight if their BMI was between 25.0 kg/m2 and 29.9 kg/ m2, obese if their BMI was 30 kg/m2 or higher, and underweight if there BMI was <18.5 kg/m2 [17].
2. *Biochemical investigations*
  - Hemoglobin level was requested for 57.9% of patients by the physician. The referred patients for hemoglobin assessment were those clinically suspected by the physician to suffer from anemia and they agreed for sampling. The hemoglobin level to diagnose anemia at sea level is less than 13 g/dl in males and less than 12 g/dl in females according to the WHO reference [18].
3. *Clinical examination*
  - The patient-generated subjective global assessment (PG-SGA) was the tool used for clinical assessment of nutritional status. It is used clinically in different settings; inpatient, outpatient, homecare, and geriatric institutes [19–21].

The Scored PG-SGA encompasses the following:

I. Patient-generated historical component which includes four items: weight history, food Intake, symptoms, activities, and function
II. Professional component which includes three items: the disease and its relation to nutritional requirements; the metabolic demand such as fever, fever duration, and corticosteroid use; and the physical examination which includes loss of subcutaneous fat, muscle wasting and edema (e.g., ankle or sacral), or ascites.

PG-SGA global assessment categories are as follows:

- *Stage A (well-nourished)*. No weight loss, recent non-fluid weight gain, no nutrient intake deficit, significant recent improvement of symptoms allowing adequate intake, no functional deficit, significant recent improvement of function, and no physical deficit or chronic physical deficit but with clinical improvement.
- *Stage B (moderately malnourished)*. Weight loss less than 5% within 1 month or 10% in 6 months, no weight stabilization nor weight gain, definite decrease in nutrient intake, presence of nutrition-related symptoms, moderate functional deficit, recent deterioration of function, mild to moderate loss of subcutaneous fat and/or muscle mass, and/or muscle tone on palpation.
- *Stage C (severely malnourished)*. Weight loss more than 5% within 1 month or more than 10% in 6 months, no weight stabilization nor weight gain, severe deficit in nutrient intake, presence of nutrition impact symptoms, severe functional deficit, recent deterioration of function or evidence of severe loss of subcutaneous fat, and possible edema [22].

Nutritional triage recommendations are as follows:

The continuous additive score is used to specify the nutritional interventions suitable for each patient [22]. Those interventions have a spectrum from patient and family education, symptom management including pharmacologic intervention to nutrient intervention such as food, nutritional supplements, enteral, and parenteral nutrition.

0–1: No intervention required. Reassess on routine and regular basis during treatment.

2–3: Patient and family education.

4–8: Requires intervention by dietitian.

≥ 9: Indicates critical need for improved symptom management and/or nutrient intervention options [22].

Assessment of dietary intake are as follows:

1. Dietary habits questionnaire
  - The dietary habits questionnaire included the number of meals per day, the main meal, breakfast intake, outdoor fatty meals intake, types of snacks, method of cooking meat, method of cooking vegetables, and types of fats used in cooking [23, 24]
2. Semi-quantitative food frequency questionnaire
  - The semi-quantitative food frequency questionnaire included 32 food items grouped into food groups: grains, vegetables, fruits, meat, milk and dairy products, beans, oils, and sugars. The patients were asked about the frequency of consumption of each food item (how many times per day or week or month and the amount consumed in household measures) over the previous year; this was subsequently converted to daily servings. The portions of food eaten from each group were calculated and compared to recommended dietary allowance (RDA) (Food and Agriculture Organization (FAO) and the World Health Organization (WHO) [25] and the National Nutrition Institute (NNI) [26]).

#### Assessment of fatigue

The Modified Fatigue Impact Scale 5-items version (MFIS-5) was used. The MFIS-5 measures the impact of fatigue on cognitive, physical, and psychosocial function which are considered by some authors to be three important sub-scales in patients with MS [27]. Patients are asked to choose one number (from a 5-point Likert scoring system) that best indicates how often fatigue has affected them during the past 4 weeks. The scale ascends from “never”, “rarely”, “sometimes”, “often”, and “almost always” each scored 0–4, respectively. Five statements are offered. The sum provides a total score from 0 to 20. The higher scores indicate greater fatigue.

### Statistical analysis

The pre-coded data were entered and analyzed using the statistical package SPSS version 21 [28]. Categorical variables were expressed in percentages. Continuous variables were expressed as range, mean ± standard deviation, and median. Chi-square test, t-test, and the one-way analysis of variance (one-way ANOVA) were applied as appropriate. *P* < 0.05 was considered statistically significant. Nutrient intake analysis was carried out for the different macro and micronutrients using the National Nutrition Institute food composition tables for Egyptian foods [29]. As regards adequacy, nutrient intake was classified according to the level of consumption compared to the FAO, WHO, and United Nations University (UNU) 2001 human energy requirements and the FAO and WHO 2001 human vitamin and mineral requirements, where < 50% was considered unsafe level of consumption, > 50–75% considered needs improvement, > 75–120% considered acceptable level of consumption, and > 120% considered over consumption [25, 30].

### Ethical considerations

The Ethical Review Committee in the Faculty of Medicine at Cairo University revised and approved the study protocol. The committee approval code was N-10-2017. All the included patients were treated according to Helsinki Declaration of biomedical ethics [31]. Informed consent from each patient was obtained after proper orientation regarding the objectives of the study. Data confidentiality and informants’ identity were maintained throughout the study and completed questionnaires were coded and accessed by the investigators only.

## Results

The present study included 76 eligible patients who attended the Kasr Alainy Multiple Sclerosis Unit (KAMSU) during the study period. The mean age of the enrolled patients was 30 ± 6 years, ranged between 20 and 40 years. The majority were females (81.6%) and 60.5% were married. More than half (51.4%) were university graduates (46.1%) or postgraduates (5.3%). Sixty percent of the patients lived in slum areas. More than three quarters of the patients (76.3%) were not working. According to El-Gilani socioeconomic scoring system, about half of the patients (56.6%) were of middle socioeconomic class. More than one third were of very low or low socioeconomic class. The median disease duration was 45 ranged from 2 to 264 months. The median Modified Fatigue Impact Scale 5-items version (MFIS-5) was 12 and ranged from 2 to 18.

**Fig. 1:**
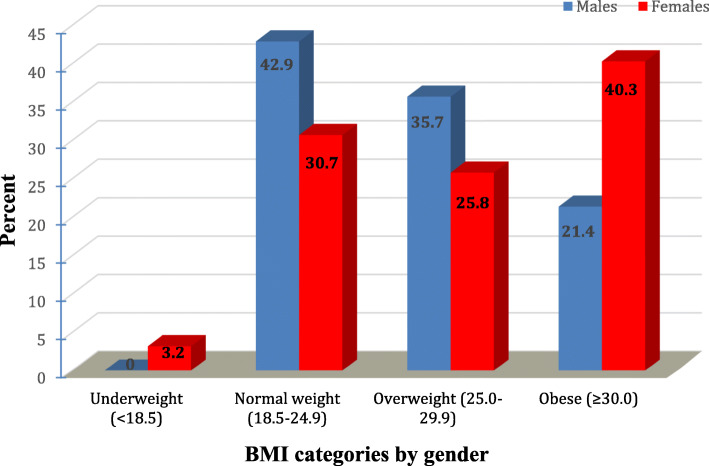
Percent distribution of the studied multiple sclerosis patients at Kasr Al-Ainy Hospital according to BMI categories and gender (n = 76)

About two thirds of women (66.1%) and 57.1% of men had excess body weight. More males were overweight (35.7% compared to 25.8% of females) and more females were obese (40.3% compared to 21.4% of males). Underweight was only observed among females (3.3%) as shown in Fig. 1.

**Fig. 2:**
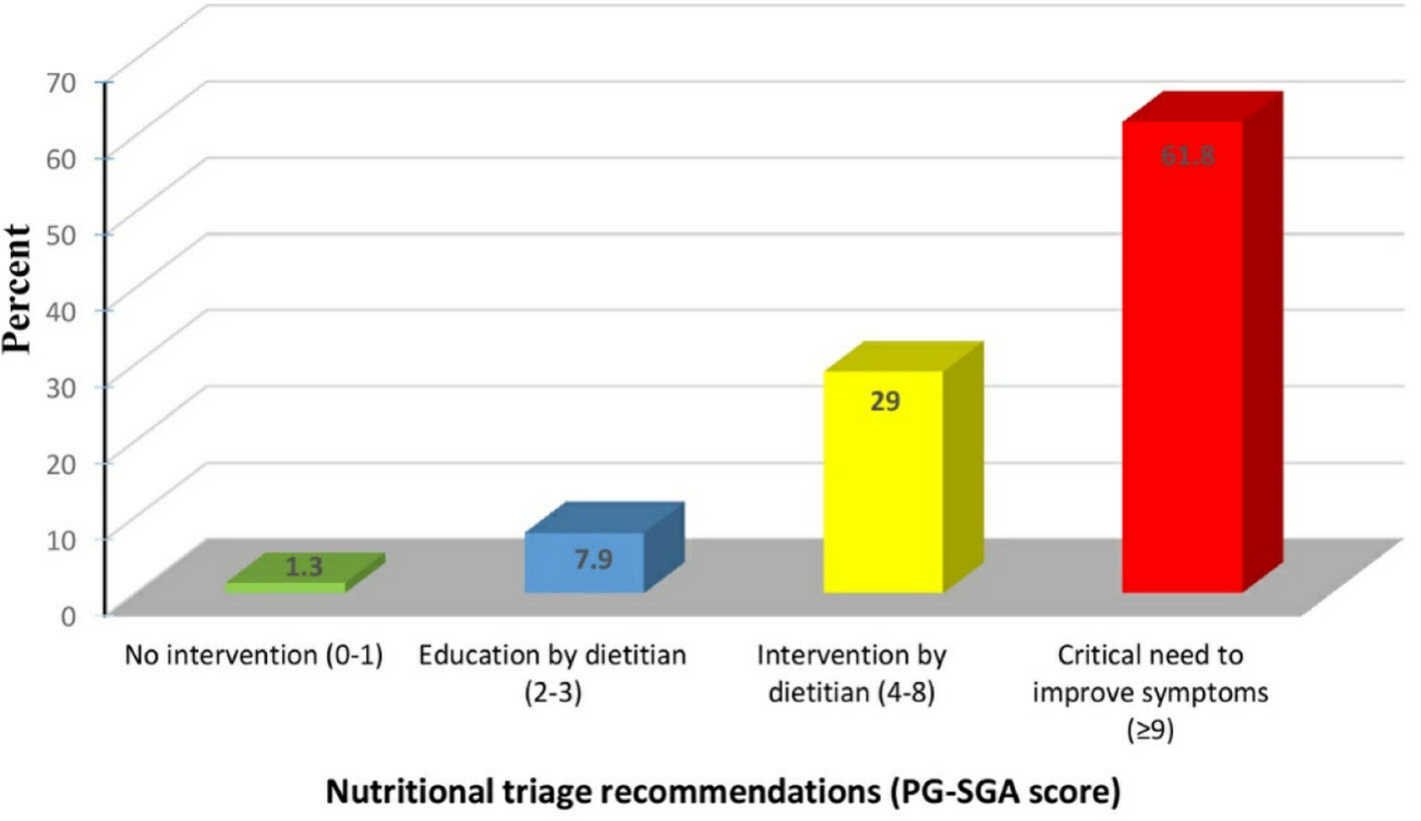
Nutritional triage recommendations according to the patient-generated subjective global assessment (PG-SGA) score of the studied multiple sclerosis patients at Kasr Al-Ainy Hospital

According to the PG-SGA, more than half of the studied patients (53.9%) were classified as moderately or suspected malnourished. The majority of patients who had normal weight (72.0%) were classified as well-nourished by the PG-SGA. All the underweight patients (100%) were classified as moderately or suspected malnourished by the PG-SGA. The association between the BMI and PG-SGA was statistically significant (*p* = 0.01) as depicted in Table 1. Figure 2 shows that about two thirds (61.8%) of patients had PG-SGA score ≥ 9, while more than a quarter (28.9%) had PG-SGA score 4–8.

**Table 1:** Percent distribution of the studied multiple sclerosis patients body mass index and patient-generated subjective global assessment categories

PG-SGA categories	BMI^a^ categories										***p-***value
	Overall, *n* = 76		Underweight, *n* = 2		Normal, *n* = 25		Overweight, *n* = 21		Obese, *n* = 28		
	No.	%	No.	%	No.	%	No.	%	No.	%	
Well-nourished	35	46.1	0	0.0	18	72.0	7	33.3	10	35.7	0.01*
Moderate or suspected malnutrition	41	53.9	2	100.0	7	28.0	14	66.7	18	64.3	

^a^Body mass index

*Significant *p-*value; N.B. no cases were severely malnourished

**Fig. 3:**
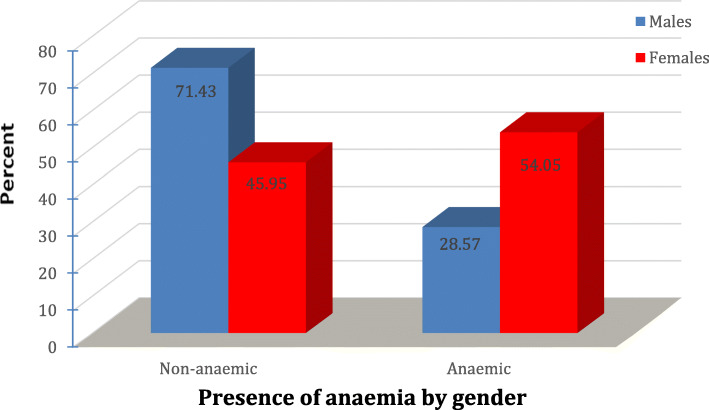
Percent distribution of the studied multiple sclerosis patients at Kasr Al-Ainy Hospital by gender and presence of anemia (n = 76)

Half (50%) of the tested patients were anemic. The latter affected more than half (54.1%) of females and more than one quarter (28.6%) of males. The observed difference, however, was statistically insignificant (*p* = 0.42) (Fig. 3).

Regarding their dietary habits, Table 2 shows that more than half of the patients (51.3%) had three main meals or more per day. Lunch was reported as the main meal by about two thirds (63.2%) of the patients. The majority of the patients (71.1%) took breakfast. About a quarter consumed fast foods. Meal characteristics did not differ significantly between BMI categories (*p* ˃ 0.05). The most frequently consumed snacks were fresh fruits and vegetables, fresh juices, and nuts or seeds (88.2%, 40.8%, and 75%) of cases respectively. Crackers and cakes were eaten as snacks by more than 70% of patients. No statistically significant difference was observed between the different BMI categories (*p* ˃ 0.05) except for butter use where the higher proportion of overweight patients used butter in cooking (*p* = 0.02).

**Table 2:** Distribution of the studied multiple sclerosis patients at Kasr Al-Ainy Hospital by dietary habits and BMI

Dietary Habits	BMI^a^ categories										***p-***value
	Overall, *n* = 76		Underweight, *n* = 2		Normal, *n* = 25		Overweight, *n* = 21		Obese, *n* = 28		
	No.	%	No.	%	No.	%	No.	%	No.	%	
**Meal number**											
- One	2	2.63	0	0.0	0	0.0	0	0.0	2	7.14	0.71
- Two	34	44.74	1	50.0	12	48.0	8	38.1	13	46.43	
- Three or more	40	52.63	1	50.0	13	52.0	13	61.9	13	46.43	
**Main meal**											
- Breakfast	13	17.1	0	0.0	3	12.0	5	23.8	5	17.9	0.06
- Lunch	48	63.2	0	0.0	16	64.0	15	71.4	17	60.7	
- Dinner	15	19.7	2	100.0	6	24.0	1	4.8	6	21.4	
**Breakfast**											
- Yes or sometimes	54	71.1	2	100.0	21	84.0	15	71.4	16	57.1	0.139
- No or rarely	22	28.9	0	0.0	4	16.0	6	28.6	12	42.9	
**Fast food intake**											
- Yes	21	27.6	0	0.0	8	32.0	7	33.3	6	21.4	0.6
- No	55	72.4	2	100.0	17	68.0	14	66.7	22	78.6	
**Snacks**											
- Fresh fruits and vegetables	67	88.2	2	100.0	21	84.0	20	95.2	24	85.7	0.6
- Fresh juices	31	40.8	2	100.0	11	44.0	12	57.1	6	21.4	0.02*
- Nuts and seeds	57	75.0	1	50.0	21	84.0	13	61.9	22	78.6	0.28
- Crackers	55	72.4	0	0.0	19	76.0	15	71.4	21	75.0	0.14
- Cakes	60	78.9	2	100.0	20	80.0	17	81.0	21	75.0	0.83
- Soft drinks	49	64.5	0	0.0	19	76.0	14	66.7	16	57.1	0.12
- Sweets	61	80.3	2	100.0	20	80.0	17	81.0	22	78.6	0.91
- Hot caffeinated drinks	68	89.5	2	100.0	20	80.0	20	95.2	26	92.9	0.3
**Cooking methods**											
**Cooking meats**^b^											
- Boiled	47	61.8	2	100.0	14	56.0	11	52.4	20	71.4	0.32
- Grilled	44	57.9	1	50.0	15	60.0	11	52.4	17	60.7	0.93
- Fried	61	80.3	2	100.0	19	76.0	16	67.2	24	85.7	0.68
**Cooking vegetables**^b^											
- Cooked with fat	56	73.7	1	50.0	22	88.0	14	66.7	19	67.9	0.24
- Boiled	30	39.5	1	50.0	9	36.0	7	33.3	13	46.4	0.77
**Cooking fat type**^b^											
- Butter	60	78.9	2	100.0	21	84.0	20	95.2	17	60.7	0.02*
- Hydrogenated oil	37	48.7	1	50.0	13	52.0	7	33.3	16	57.1	0.41
- Oil	71	93.4	2	100.0	23	92.0	19	90.5	27	96.4	0.42

^a^Body mass index

^b^More than one response was allowed

*Significant *p-*value

No statistically significant difference was observed between the different BMI groups (*p* ˃ 0.05) except for fresh fruit juices intake (*p* = 0.02) where the lower proportion of obese patients consumed fresh fruit juices.

The majority (80.3%) of the patients fried the meats, 73.7% cooked the vegetables with fats, 93.4% used oils, and 78.9% used butter in cooking.

Regarding their dietary intake, Table 3 shows that the majority (84.2%) of the studied patients overconsumed proteins. All the studied patients (100%) overconsumed fats. The total daily caloric consumption was unsafe in 2.6% and needed improvement in 19.7% of the studied patients. On the other hand, the majority of the patients (89.5%) unsafely consumed the dietary fibers.

**Table 3:** The daily caloric and macronutrients’ intake from diet among the studied multiple sclerosis patients at Kasr Al-Ainy Hospital

Nutrient	The daily intake							
	Unsafe < 50%		Needs improvement 50–75%		Acceptable 75–120%		Over consumption ≥ 120%	
	No.	%	No.	%	No.	%	No.	%
■ **Energy (Kcal)**	2	2.63	15	19.74	41	53.95	18	23.68
■ **Proteins**	0	0.0	1	1.3	11	14.5	64	84.2
■ **Total fats**	0	0.0	0	0.0	0	0.0	76	100.0
■ **Dietary fibers**	68	89.5	6	7.9	2	2.6	0	0.0

According to the semi-quantitative food frequency questionnaire

Table 4 shows that all the studied patients (100%) suffered from fatigue. The vast majority (93.4%) reported physical fatigue. Cognitive and psychological fatigue were reported by 76.3% and 60.5% of the patients.

**Table 4:** Prevalence of physical, cognitive, and psychological fatigue among the studied multiple sclerosis patients at Kasr Al-Ainy Hospital by gender

Fatigue type	Gender						***p-***value
	Overall, *n* = 76		Male, *n* = 14		female, *n* = 62		
	No.	%	No.	%	No.	%	
■ **Physical fatigue**	71	93.4	13	92.9	58	93.5	0.93
■ **Cognitive fatigue**	58	76.3	9	64.3	49	79.0	0.24
■ **Psychological fatigue**	46	60.5	9	64.3	37	59.7	0.75
■ **Fatigue**	76	100.0	14	100.0	62	100.0	-

## Discussion

The current study revealed that about two thirds (64.5%) of the participating relapsing-remitting multiple sclerosis patients were overweight or obese. In addition, almost all of the patients had PG-SGA scores which indicated their need of nutritional intervention. The semi-quantitative food frequency questionnaire revealed overconsumption of energy and fat, while the majority of the studied patients consumed dietary fibers in less than the acceptable levels.

The prevalence of overweight and obesity among MS patients was 64.5%, which is close to the prevalence reported among American MS patients (60%) [8], but it was higher than that observed among Turkish MS patients (40.5%) [32]. This might be attributed to the unsound dietary habits of the studied patients since about two thirds of them (61.8%) consumed bakeries with high fat, sugar, and refined wheat content and Egyptian pie 3–6 times/week.

Half of the current study participants were anemic. The prevalence of anemia in the studied group was much higher than that reported in Rome, Italy (18.7%), and in Iran (18.6%), considering that the Iranian study cutoff level for anemia diagnosis was higher than our cutoff level by 0.5 g/dl. This might be explained by the fact that the hemoglobin level was tested for those clinically suspected to be anemic not all the studied patients by the treating physician. Females had double the prevalence in males in both the current study and the Italian one; however, this difference was not statistically significant in any of these studies [33, 34].

According to the PG-SGA, about half of the patients had moderate or suspected malnutrition. This could be explained by the current findings that revealed that the majority of the patients had nutrition-related symptoms that kept them from eating during the past 2 weeks before the interview such as depression, dry mouth, nausea, dental, swallowing, and financial problems. More than half (56.6%) and about a third (35.5%) of the patients reported suffering from dry mouth and swallowing problem respectively. Similar rates were reported in a study conducted in the College of the Medicine University of Wales, UK (n = 79), in which 51.9% and 34.2% of the patients reported suffering from dry mouth and swallowing problem respectively [35]. Almost all patients required some form of nutritional intervention while actually nothing is being done. In spite of the high prevalence of these symptoms, underweight affected only 2.6% of the studied patients. Weight loss is known to occur in advanced MS. It may be due to decreased dietary intake, dysphagia, and depression. When disability advances, weight loss, undernutrition, and cachexia occur [9]. In our study, patients were at an early stage of the disease (RRMS), the median disease duration was 45 ranged from 2 to 264 months. Accordingly, initiation of nutrition care service is extremely needed in the unit.

The unhealthy dietary habits such as taking only less than three meals, junk food intake, and skipping breakfast were observed in considerable proportions of the group. Although the majority of the patients consumed vegetable oils in cooking, more than three quarters (79.2%) used saturated fats (butter) which were significantly consumed more by overweight patients. Higher proportions of the patients fried the meats and cooked the vegetables with fats. Half of the patients used hydrogenated oils in cooking. Those habits were the reverse of what was recommended in a review article which stated that trans-fatty acids (TFAs) which are found in some foods namely hydrogenated oils, fried food, and junk food and in snacks must be controlled to avoid the rise of inflammatory processes in MS [1].

Healthy dietary habits such as intake of nuts, fish, fruits, and beans were less frequent in the patients of the current study than the patients of a study conducted in Ahvaz, Iran [35]. Also, patients in the current study consumed hydrogenated oils and meat less frequent than the patients of the Iranian study. This may be attributed by the socioeconomic or the cultural differences between the two groups. Since unemployment was prevalent among the studied patients, their income was affected. The majority (88.0%) of the studied patients who stated that they were in debt were unemployed.

About half of the studied patients consumed milk on daily basis. All but one of them chose the full cream types. Consumption of unskimmed milk, animal fat, and smoked meat were nutritional risk factors of primary demyelination in a high-risk area for multiple sclerosis [36]. Also, Riccio and Rossano stated that patients with MS should avoid the intake of whole milk and prefer skimmed milk, because cross-reactive antibodies between the components of the whole milk and the human CNS have been found in MS patients [1]. More than a quarter of the studied patients consumed pickles daily. Kleinewietfeld mentioned that increased dietary salt intake might be an environmental risk factor for the development of autoimmune diseases by inducing pathogenic cells and related proinflammatory cytokines [37]. Those cells have been involved in the development of MS [1].

### Conclusion

The study concluded that overweight, obesity, anemia, and bad dietary habits are prevalent among the RRMS patients attending the KAMSU. Nutritional care should be an integral part of the management of this group of patients.

### Study limitations

The current study findings should be viewed with respect to the following limitations: The descriptive nature of the study. It was conducted to explore the situation in this new area of inquiry and to generate hypotheses as no information is available regarding the nutritional status of RRMS patients in Egypt. It was not used to infer causal relationships. The semi-quantitative food frequency questionnaire may have some weaknesses such as recall bias, inaccurate estimation of portion sizes, and possible over/under-reporting of certain foods (e.g., fruit and vegetables). One of the researchers who is a European Society of Parenteral and Enteral Nutrition (ESPEN)-qualified clinical nutrition specialist conducted all interviews herself to ensure good quality of data. Also, the semi-quantitative food frequency questionnaire was complemented by other tools of nutritional assessment such as the anthropometric, biochemical, and clinical tools.

## Data Availability

The datasets used and/or analyzed during this study are available from the corresponding author on reasonable request.

## Abbreviations

RRMS: Relapsing-remitting MS
KAMSU: Kasr Alainy Multiple Sclerosis Unit
RDA: Recommended dietary allowance
FAO: Food and Agriculture Organization
WHO: World Health Organization
NNI: National Nutrition Institute
UNU: United Nations University
